# Experimental Investigation of Al_2_O_3_ Nano-Powder-Mixed Dielectric in EDM-Assisted Micro-Milling

**DOI:** 10.3390/mi16070725

**Published:** 2025-06-21

**Authors:** Sharad Yadav, Deepak Agarwal, Anuj Kumar Sharma, Rabesh Kumar Singh, Saurabh Chauhan, Shalini Mohanty

**Affiliations:** 1 Centre for Advanced Studies, Dr. A.P.J. Abdul Kalam Technical University, Lucknow 226031, Uttar Pradesh, India; sharadyadav78654@gmail.com (S.Y.); deepagg23me@gmail.com (D.A.); anujksharma@cas.res.in (A.K.S.); 2 Department of Mechanical Engineering, IET, Dr. Ram Manohar Lohia Avadh University, Ayodhya 224001, Uttar Pradesh, India; 3 Mechanical Engineering Department, Madan Mohan Malaviya University of Technology, Gorakhpur 273010, Uttar Pradesh, India; 4 Applied Science and Humanities, Rajkiya Engineering College, Kannauj 209732, Uttar Pradesh, India; 5 Faculty of Engineering and Sciences, University of Greenwich, Chatham Maritime ME4 4TB, UK

**Keywords:** micro-EDM, powder-mixed μ-EDM, alumina, micro-milling, micro-channel, DFA

## Abstract

This paper investigates the use of Al_2_O_3_ nano-powder-stirred micro-EDM process for generating micro-channels. This study focuses on the effect of critical machining process parameters, such as capacitance levels and nano-powder concentration, on the micro-channel fabrication performance in terms of TWR, MRR, depth, and width. A two-stage nested ANOVA is employed to understand the effect of powder concentration within different capacitance levels. The results show that the powder concentration significantly influences the system’s performance in conjunction with the capacitance. At low (100 pF) and high (1000 pF) capacitance, the addition of Al_2_O_3_ nano-powder increases the MRR, depth, and width but decreases TWR up to a concentration of 1.0 g/L. A desirability function analysis (DFA) tool identified the best overall performance from 14 experiments, revealing that 100 pF and 1 g/L yield the optimal outcomes.

## 1. Introduction

**Figure 1:**
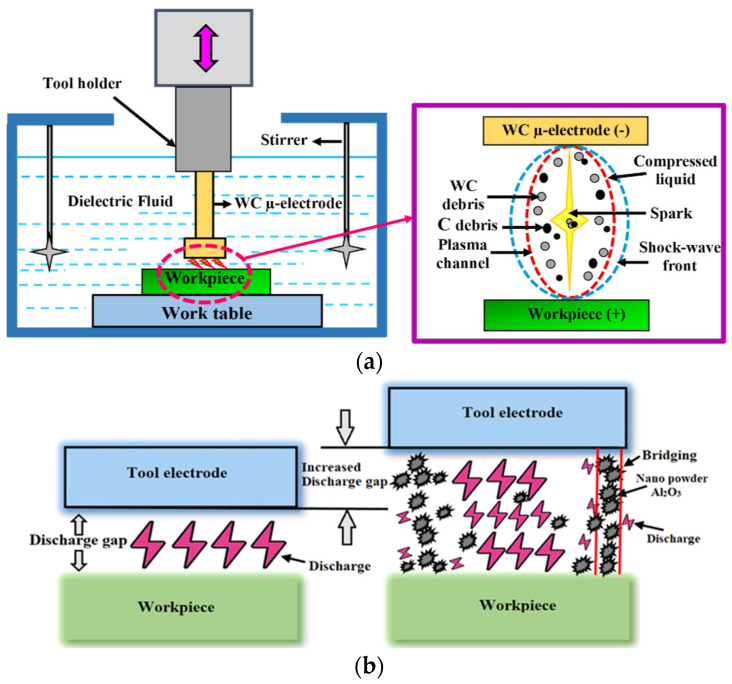
(**a**) Illustration of traditional micro-EDM and spark generation. (**b**) Spark generation with and without powder-mixed micro-EDM.

Micro-EDM is generally used for producing precise micro-sized components or parts, making it an essential tool in micro-level manufacturing [1]. The demand for miniaturized parts has expanded in fields such as microelectronics, biomedical devices, precision engineering, military applications, and aerospace [2]. As a result, various microfabrication methods have been developed, including micro-EDM, which can produce miniature components ranging from micrometers to a few millimeters in size [3,4]. Researchers have worked extensively to create simpler, low-cost technologies for producing micro-scale devices [5]. Micro-EDM can create parts measuring 1–500 µm, thus achieving finished products on the micron scale [6]. This process uses spark discharges between a small-diameter conductive tool electrode and a workpiece, removing materials by melting and vaporizing when an electrical pulse is applied [7]. Figure 1a illustrates the electrical discharge and spark gap phenomenon in micro-EDM [8,9,10,11]. Micro-EDM holds the potential to precisely create µ-features like micro-holes and micro-slots/channels with dimensions as small as a few micrometers in size [9,12]. Micro-channels are broadly used in microfluidic devices (e.g., miniature heat exchangers, micro-reactors, and micro-fuel cells) for manipulating fluid flow [13,14,15]. These devices have a wide range of applications in chemical analysis, biological sensing, drug discovery, and lab-on-a-chip technology [16]. They also enhance heat transfer in electronics (e.g., microprocessors) and serve in micro-electromechanical systems (MEMSs), sensors, micro-heat exchangers, and aerospace components [17,18,19].

Several research attempts have unfolded to enhance the performance of micro-EDM machining. One of the most common techniques for improving the surface integrity and metal removal rate is powder-mixed micro-EDM [20,21]. In the powder-blended micro-EDM technique, a small amount of nano-powder is stirred in a dielectric. When nano-powder is dispersed in the dielectric, the particles become polarized and assist in forming conductive bridges, promoting easier dielectric breakdown and improving stability [22,23,24]. Figure 1b shows the bridging effects and differences in gap phenomena between traditional micro-EDM and powder-mixed micro-EDM (PM micro-EDM). Several studies indicate that adding nano-powder enhances the machining efficiency by raising the spark frequency, which can increase MRR and reduce TWR [25,26,27,28,29]. Kumar et al. [30] mixed alumina nano-powders into deionized water as a dielectric for EDM drilling and observed significantly improved surface finish relative to conventional EDM. Tan et al. [31] employed SiC and Al_2_O_3_ nano-powders during micro-EDM drilling and achieved better surface quality. Prihandana et al. [32] found that nano-sized MoS_2_ powder performed better than micro-sized MoS_2_ in die-sinking micro-EDM. Sivaprakasam et al. [33] used nano-graphite in µ-Wire-EDM on nickel alloy-625, observing improved surface quality.

**Figure 2:**
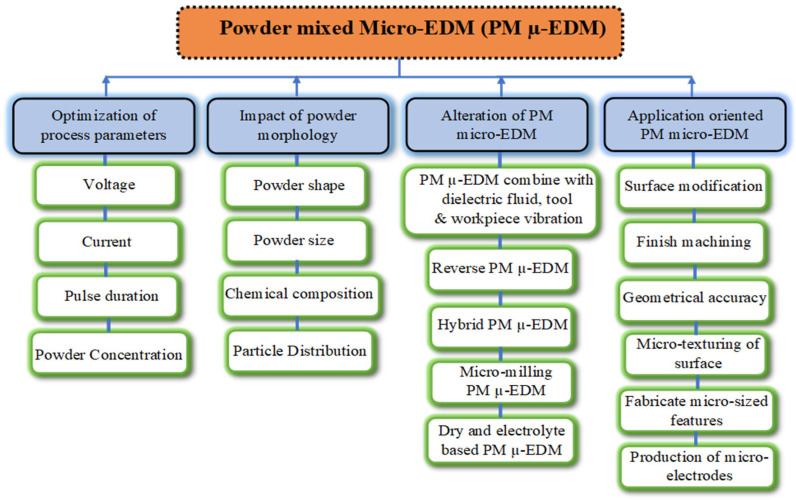
Methods to improve the machine performance using powder-mixed micro-EDM.

Mohanty et al. [34] used the particle swarm optimization and response surface techniques to analyze the results of AlSiCp composites using Al_2_O_3_ nano-powder-mixed EDM. The experimental results revealed that using Al_2_O_3_ nano-powder enhances the machining rate and surface quality. Elumalai B et al. [35] found that adding Al_2_O_3_ nano-powder reduced micro-crack formation and crater size while increasing MRR and TWR. Furthermore, Kuriachen et al. [36] also demonstrated that SiC nano-powder enhances MRR and modifies the machined surface in the micro-EDM milling of Ti-6Al-4V. In the past, numerous researchers have examined the effect of nano-powder-stirred micro-EDM on various aspects, such as optimization of process parameters, influence of powder morphology, surface modification, and application-oriented NPMEDM, as shown in Figure 2. Figure 2 illustrates various techniques used to improve the performance of powder-mixed micro-EDM. These methods include selecting the appropriate powder materials, adjusting the powder concentration, optimizing the discharge energy levels, applying cryogenic or ultrasonic assistance for uniform dispersion, and employing statistical and AI-based optimization approaches. The figure serves as a roadmap, summarizing how material selection and process innovation collectively contribute to enhanced MRR, reduced TWR, better dimensional accuracy, and superior surface finish in micro-EDM processes. Kumar et al. [37] investigated the effectiveness of Al_2_O_3_ NPMEDM and reported greater spark stability, higher MRR, and improved surface quality compared to pure dielectric EDM. Furthermore, the addition of nanoparticles significantly reduced microcracks and thermal stress compared to conventional methods. Moreover, Pillai et al. [38] examined the impact of nano-graphene on micro-EDM using a cryogenically treated WC electrode on a titanium alloy workpiece. The outcomes show that the cryogenically treated tool, with nano-graphene powder added to the dielectric, significantly improves MRR, TWR, and surface quality. Jeavudeen et al. [39] examined the effect of adding different powders, such as SiC, Cu, Al_2_O_3_, and Al, to the dielectric. Their experimental findings showed that nano-powder absorption improves MRR but reduces the dielectric strength.

Abdul-Rani et al. [40] showed that nano-aluminum powder enhances the surface integrity by minimizing cracks and voids. Sahu and Mandal [41] found that graphite-mixed PM micro-EDM improves the surface finish and dimensional tolerance while reducing the recast layer thickness. Additionally, Jahan et al. [42] emphasized that powder-mixed EDM milling can produce smoother, defect-free surfaces, while Sahu et al. [43] noted that alumina (Al_2_O_3_) nano-powder, due to its excellent dielectric and thermal properties, improves the machining efficiency and surface finish. Al₂O₃ nano-powder, being low-cost with low density, moderate thermal conductivity, and high wear resistance, is suitable for micro-EDM. Its thermal stability and insulation improve the surface finish and MRR and reduce cracks, voids, and the recast layer. It also ensures a stable discharge with minimal chemical interaction compared to SiC, MoS₂, and graphite [44]. Its use can locally increase the temperature at the spark site, enhancing material softening and removal and reducing the machining time. Although prior work has explored aspects of powder-mixed micro-EDM, fewer studies have addressed the combined effect of nano-powder concentration under high and low capacitance conditions for micro-channel fabrication. Therefore, the present study investigates how Al_2_O_3_ nano-powder concentration influences the MRR, TWR, width, and depth of micro-channels, focusing on low (100 pF) and high (1000 pF) capacitance levels. This work applies a two-stage nested ANOVA and a desirability function analysis (DFA) to determine the optimal parameters. The generated micro-channels are analyzed for their dimensional accuracy (depth and width), material removal rate, tool wear rate, and surface morphology via scanning electron microscopy (SEM) and energy-dispersive X-ray spectroscopy (EDX).

## 2. Materials and Methods

### 2.1. Electrode and Workpiece Material

Copper is frequently used in manufacturing heat sinks and micro-channels for medical devices due to its excellent thermal conductivity and biocompatibility. It is also used in electronic components to improve performance [18]. The present work uses a copper sheet (40 mm × 20 mm × 2 mm) as workpiece material to generate micro-channels using the micro-EDM process. A cylindrical-shaped tungsten carbide (WC) tool material with a diameter of 500 µm is utilized as the electrode. The tool and electrodes were procured from PK Enterprises, Dhanbad, India. Table 1 illustrates the physical characteristics of both the tool electrode and the workpiece materials.

### 2.2. Experimental Setup

**Figure 3:**
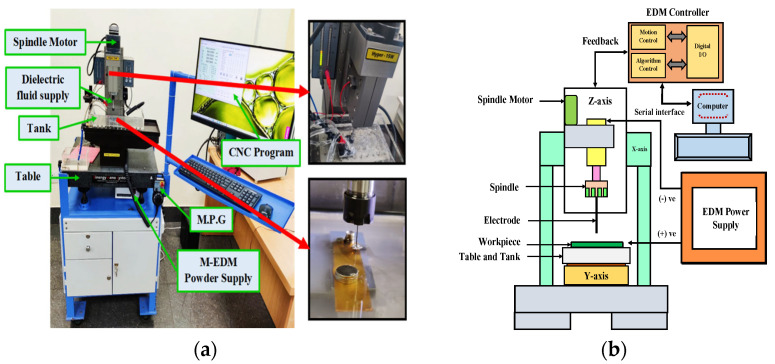
(**a**) Complete and close view of experimental setup (model: Hyper 15 Micro-EDM, make: Sinergy Nano Systems in Navi Mumbai, India). (**b**) The schematic diagram shows the experimental test setup.

All experiments were performed on a tabletop micro-EDM machine (model: Hyper 15 Micro-EDM, Sinergy Nano Systems, Navi Mumbai, India) equipped with an RC-type pulse generator, as shown in Figure 3a. This tabletop micro-EDM machine can perform various fabrication techniques, like micro-EDM, µ-milling, µ-drilling, µ-Wire EDM, and µ-electric discharge grinding, with additional control and accessories. Several pilot tests confirmed the consistency of responses and helped to determine the parameters for the main experiments.

Commercial EDM oil served as the dielectric fluid. A stirrer and a probe-type ultrasonicator were used to mix Al_2_O_3_ nano-powder (25 nm, spherical) at different concentrations (0.25 to 2.5 g/L). From pilot tests and the literature, smaller nanoparticles generally enhance MRR and reduce TWR [23]. Figure 3a shows the spindle-tool assembly, and Figure 3b depicts the schematic layout. The depth and volume of generated micro-channels were measured with a non-contact 3D optical surface profilometer (Model: NewView TM 9000, Zygo Corporation, Middlefield, OH, USA), while an OLYMPUS (Tokyo, Japan) Moticam 5+ optical microscope measured the channel width. A ZEISS (Oberkochen, Germany) Gemini SEM 300 field emission SEM (FESEM) with EDX was used to analyze the machined surfaces. Table 2 shows the machine’s technical specifications.

### 2.3. Machining Parameters

Factors that affect the machining performance of micro-EDM include work material, electrode material, capacitance, voltage, feed rate, dielectric medium, powder concentration, tool speed, polarity, etc. There are several parameters that affect the machining performance, but, based on some pilot tests, capacitance and nano-powder concentration are selected for the present study. Table 3 details the chosen parameters and levels. The voltage, feed rate, and slot dimensions were held constant. Four performance measures, i.e., MRR, TWR, depth, and width, were recorded.

### 2.4. Design of Experiments

**Figure 4:**
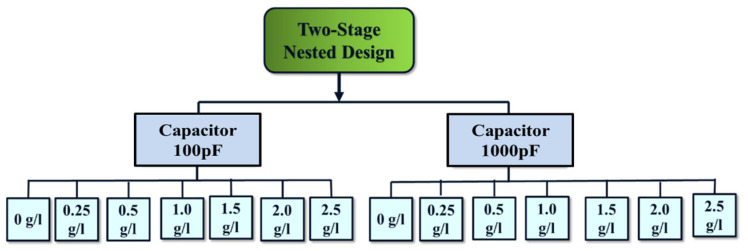
The flowchart shows the DOE using a two-stage nested design.

The statistical analysis is evaluated by a two-stage nested design to analyze the experimental data, in which there are two levels of nested factors. This method is effective for assessing the influence of multiple factors on the output responses. In the present work, the two-stage nested design is applied to the micro-EDM process to investigate the impact of various machine parameters on the machining accuracy and surface finish. The analysis proceeds in the following two steps: firstly, by evaluating the differences among the capacitance levels; and secondly, by examining the differences among the powder concentration levels used during the micro-EDM machining process. The experimental design, based on the two-stage nested structure, is illustrated through the tree diagram shown in Figure 4. The data recorded during different experiments are analyzed using a two-stage nested ANOVA analysis.

A two-stage nested ANOVA analysis is used to identify the factor that has a stronger influence on the response variable. Additionally, the interaction between the two nested factors is also examined. The linear statistical model corresponding to the two-stage nested design is presented in Equation (1).

(1) $$Y_{ijk}^{*}=\;\mu^{*}+\tau_{i}^{*}+\beta_{j(i)}^{*}+\in_{\left(ij\right)k}^{*}\left\{\begin{array}{c} i=1,2,\ldots ,a_{0}\\ j=1,2,\ldots ,b_{0}\\ k=1,2,\ldots ,n_{0} \end{array}\right.$$

The experimental design incorporates multiple levels of Factor A, with Factor B nested within each level of A. Multiple replicates are included, denoted by the subscript j(i) in Equation (1). Each level of Factor B exists only within a specific level of Factor A, and the error term is associated with replicates nested within each A and B combination, represented by the subscript (ij)k. The design is balanced, meaning that each level of A contains the same number of B levels and equal replicates.

However, there is no interaction term between A and B, as the levels of B are not common across all levels of A (i.e., incomplete nesting). Whether A and B are considered fixed or random effects influences the appropriate statistical model used for evaluating their impact. Considering that components A and B are constant, then $\sum_{i=1}^{a_{o}}{\tau^{*}}_{i}$ = 0 and $\sum_{j=1}^{b_{o}}{\beta^{*}}_{j(i)}$ = 0 (i = 1, 2,…, a_o_). The combined impact of behavior A is zero, and the collective effect of treatment B is also zero, but only when analyzed within each level of treatment A. Instead, if A and B are chosen at random, assume that τ*_i_ is NID (0, $\sigma_{\tau^{*}}^{2}$) and ${\beta^{*}}_{j(i)}$ is NID (0, $\sigma_{\tau^{*}\beta^{*}}^{2}$). It is common to come across mixed models where A is set, whereas B is arbitrary. The steps to calculate the different values are represented below in the form of Equation (2) to Equation (6).

(2) $$\mathrm{Total}\;\mathrm{sum}\;\mathrm{of}\;\mathrm{squares}\;{SS}_{T}={SS}_{A}+{SS}_{B(A)}+{SS}_{E}$$

(3) $$\mathrm{Sum}\;\mathrm{of}\;\mathrm{squares}\;\mathrm{due}\;\mathrm{to}\;\mathrm{factor}\;A\;{\mathrm{SS}}_{A}={SS}_{A}=\frac{1}{b_{o}no}\sum_{i=1}^{a}y_{i}^{2}-\frac{y_{\ldots }^{2}}{a_{o}b_{o}n_{o}}$$

(4) $$\mathrm{Sum}\;\mathrm{of}\;\mathrm{squares}\;\mathrm{for}\;\mathrm{factor}\;B\;\mathrm{within}\;\mathrm{the}\;\mathrm{levels}\;\mathrm{of}\;\mathrm{factor}\;A\;{SS}_{B(A)}=\frac{1}{n}\sum_{i=1}^{a_{o}}\sum_{j=1}^{b_{o}}y_{ij.}^{*2}-\frac{1}{b_{o}n_{o}}\sum_{i=1}^{a_{o}}y_{i}^{*2}$$

(5) $$\mathrm{Sum}\;\mathrm{of}\;\mathrm{squares}\;\mathrm{due}\;\mathrm{to}\;\mathrm{error}\;{SS}^{E}=\sum_{i=1}^{a_{o}}\sum_{j=1}^{b_{o}}\sum_{k=1}^{n_{o}}y_{ijk}^{*2}-\frac{1}{n_{o}}\sum_{i=1}^{a_{o}}\sum_{j=1}^{b_{o}}y_{ij}^{*2}$$

(6) $${SS}^{T}=\sum_{i=1}^{a}\sum_{j=1}^{b}\sum_{k=1}^{n}y_{ijk}^{2}-\frac{y^{*2}}{a_{o}b_{o}n_{o}}$$

### 2.5. Desirability Function Analysis (DFA)

**Figure 5:**
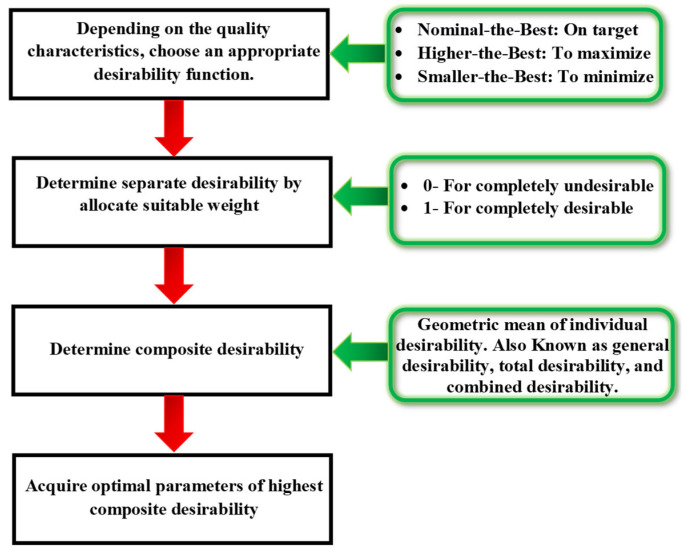
Flowchart for optimization DFA (desirability function analysis).

DFA is an analytical method acknowledged in engineering and manufacturing for optimizing multiple response variables simultaneously [45]. In micro-EDM, DFA can be useful to determine the best combination of machining parameters that result in desirable machining outcomes [6]. Harrington first introduced the concept of DFA, which was then made widely known by Derringer and Suich [46]. DFA involves constructing a desirability function that combines the desirability scores of each response variable into a single overall score. The desirability scores are calculated based on the desired target values and the acceptable limits for each response variable. The algorithm is then tuned to identify the machining settings that yield the best overall desirability score. The individual desirability index is generated when all the quality attributes are transformed to reside in the range of 0 to 1. To calculate the composite desirability index, the weighted geometric mean of each combination of answer variables is employed. The optimal parameter choices that result in the most desired quality features under evaluation are those found in datasets with the highest composite desirability. Figure 5 depicts a flowchart of the subsequent phases in the optimization process using DFA.

### 2.6. Estimation of MRR and TWR, Micro-Channel Width, and Depth

**Figure 6:**
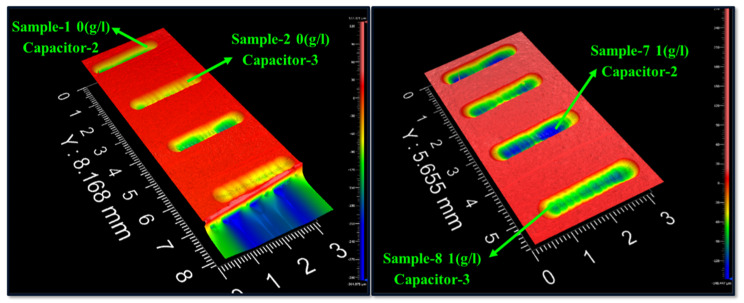
Three-dimensional images of µ-channel at different energy levels, 0 and 1 g/L powder concentration.

This experiment aims to determine the influence of machining parameters on MRR, TWR, micro-channel width, and depth. It is always advantageous to have a higher MRR, as it results in a shorter machining time. By assessing the volume of material evacuated and dividing it by the milling time, it is feasible to determine the MRR. A non-contact 3D optical surface profilometer is used to measure the volume of metal removal. Figure 6 depicted the 3D images of a micro-channel generated in different experiments. TWR is the electrode tool’s wear or erosion rate during the machining. TWR is determined by identifying the weight of the µ-tool electrode worn down over every single milling operation, as per Equation (7). The density of the tungsten carbide tool material is 0.01563 gm/mm^3^.

(7) $$TWR\;\left({mm}^{3}/min\right)=\frac{\left(W_{i}-W_{f}\right)\;of\;electrode}{Machining\;time\;\times \;Density\;of\;electrode}\;\;\;\;\;\;\;\;\;\;\;\;\;\;\;\;\;\;\;\;\;\;\;\;\;\;\;\;\;\;\;\;\;\;\;\;\;\;\;$$

**Figure 7:**
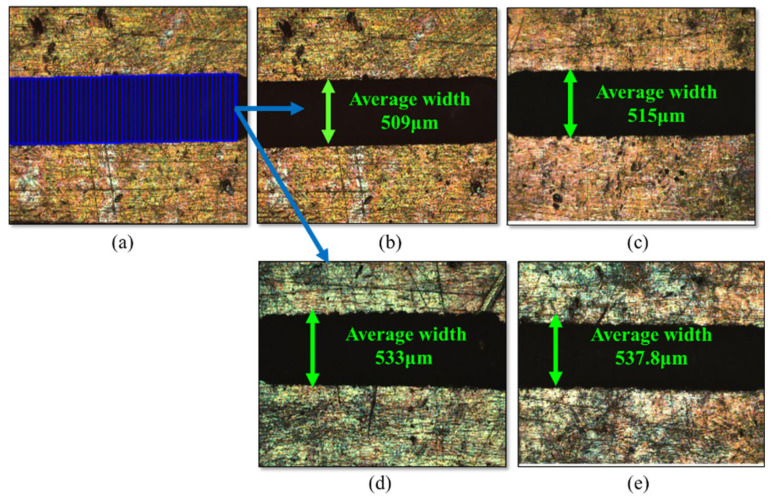
Average width of micro-channels. (**a**) Measurement of width at multiple points; (**b**) at capacitor 100 pF and 0 g/L; (**c**) at capacitor 1000 pF and 0 g/L; (**d**) at capacitor 100 pF and 1 g/L; (**e**) at capacitor 1000 pF and 1 g/L.

**Figure 8:**
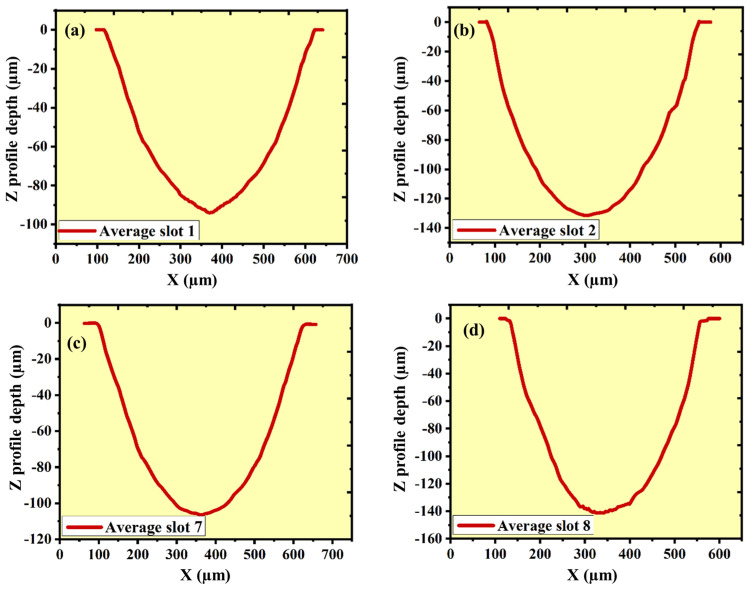
Depth profiles of micro-channel at (**a**) capacitor 100 pF and 0 g/L; (**b**) capacitor 1000 pF and 0 g/L; (**c**) capacitor 100 pF and 1 g/L; (**d**) capacitor 1000 pF and 1 g/L.

One can ascertain the micro-channel’s width by gauging the distance between the micro-channel’s walls. A metallurgical microscope (BX53, Olympus, Tokyo, Japan) is used to measure the width of the micro-channel at 10× magnification. Figure 7a shows the measurement of width at multiple places, and Figure 7b–e shows the average values of micro-width during different experiments. The depth of the µ-channel/slot can be determined using a non-contact 3D optical surface profilometer by scanning the generated surfaces. The measurement is taken at five different places in a single micro-channel, and the average of all is considered as the final value. Figure 8 depicted the depth of µ-channels at different energies and powder concentrations.

## 3. Results

All of the experiments are designed and conducted as per the two-stage nested design of experiments. The responses of the experiments are measured in terms of MRR, TWR, depth, and width of micro-channel, and the average readings are recorded as shown in Table 4. In machining, achieving minimum tool wear that prevents frequent tool changes and higher dimensional accuracies is always recommended to increase the quality of parts.

### 3.1. Two-Stage Nested Analysis

A two-stage nested analysis is used to analyze the performance characteristics of the µ-milling process in terms of TWR, micro-channel depth, MRR, and width with and without powder concentration and a capacitor. The MINITAB 21 software was used to obtain the combination of designed parameters. In a two-stage nested design, the capacitor is considered the parent factor, and powder concentration is viewed as the nested factor with different levels. An analytical analysis is carried out following examination to ascertain the impact of powder concentration and capacitor at a significant level of 5% (α = 0.05). An ANOVA analysis was performed considering the capacitor level and powder concentration level to obtain the significance of parameters on MRR, TWR, depth, and width of micro-channels. In the ANOVA analysis, it is crucial to reveal how the combined influence of nested factors is reflected through main and interaction effect plots. The step-by-step two-stage nested analysis is summarized and shown in Table 5.

The two-stage nested ANOVA analysis predicted that, alone, the powder concentration does not affect the machining performance because it acts like debris. On the other hand, the capacitor energized the nano-powder as per the concentration to create a nano-homogenized spark channel. The performance characteristics primarily depend on changes in the capacitance due to variations in the powder concentration, highlighting the significant roles of both capacitance and its interaction with the powder concentration. So, the behaviors of nano-powder concentration at different energy levels are investigated.

### 3.2. Analysis of MRR

**Figure 9:**
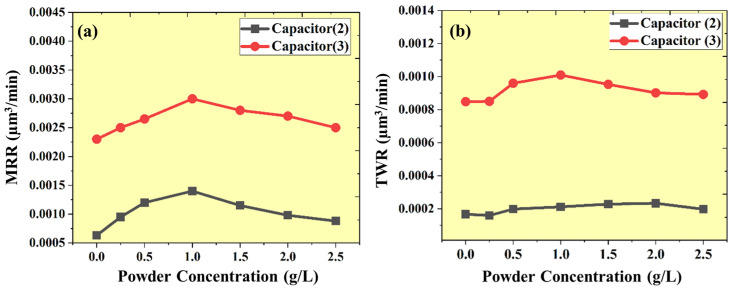
(**a**) Outcome of Al_2_O_3_ nano-powder concentration on MRR at different capacitors. (**b**) Effect of Al_2_O_3_ nano-powder concentration on TWR at different capacitors.

The interaction behavior of the capacitor and the Al_2_O_3_ nano-powder concentration on MRR is depicted in Figure 9a, which illustrates that the discharge energy enhances the effectiveness of the Al_2_O_3_ nano-powder at a particular concentration level (1.0 g/L). Beyond that, the excess powder acts as debris, which degrades the machining performance. The low capacitance levels (100 pF) are not enough to energize the nano-powder beyond 1.0 g/L concentration. So, the maximum MRR obtained was between 0.5 g/L and 1.0 g/L powder concentration. The MRR enhancement factor is high, up to 50.3%, at low capacitance levels (100 pF) due to homogenization of the discharge energy and increased gap voltage by the use of nano-powder at 0.25 g/L concentration. Increasing the powder concentration leads to a decrease in its impact on MRR. The experimentation concludes that with the powder concentration from 0.25 g/L to 0.5 g/L, the MRR is enhanced by 26.31%, and with a concentration from 0.5 g/L to 1.0 g/L, the MRR is enhanced by only 16.66%. Beyond 1.0 g/L powder concentration, the MRR is reduced by 17.85% due to powder acting as non-energized debris.

On the other hand, at a high capacitance level (1000 pF), the discharge energy is much higher to increase the MRR approx. 2.64 times in a non-uniform manner. In those conditions, the nano-powder just uniformly enhances the distribution of energy so that the dimensions will improve. This experimentation concludes that at a high capacitor level, the use of nano-powder mostly participates in the dimensional improvement and enhances the MRR by approx. only 8.7%. Increasing the concentration from 0.25 g/L to 0.5 g/L enhances the MRR by 6%, and a further increase in the concentration from 0.5 g/L to 1.0 g/L enhances the MRR by 13.6%. Beyond a powder concentration of 1 g/L, the MRR is reduced by 6.7% due to the powder acting as non-energized debris, which enhances secondary discharge shorting and arcing.

### 3.3. Analysis of Tool Wear Rate (TWR)

Since the spark series emits excessive heat, the substance is eliminated by melting and evaporating during micro-EDM. In addition to the intense heat produced when a material evaporates from a workpiece, there is also substance erosion from the tool electrode. However, at a low capacitance level (100 pF), the nano-powder promotes gap voltage, better flushing, and distributes the energy uniformly. Therefore, with the use of nano-powder, TWR will be reduced by 4.2% at a powder concentration of 0.25 g/L. Furthermore, with an increase in the concentration from 0.25 g/L to 0.5 g/L, 1.0 g/L, and 1.5 g/L, the secondary discharge at the side wall is increased. So, it promotes the erosion at the tool electrode by 24.4%, 32.5%, and 42.5%, respectively. On the other hand, at a high capacitance level (1000 pF), the nano-powder only enhances the energy distribution throughout the surface. So, TWR is reduced by 2.3%, 4.1%, and 6.8% at 0.25 g/L, 0.5 g/L, and 1.0 g/L, respectively. Furthermore, with an increase in the concentration beyond 1.0 g/L, the secondary discharge frequency enhances the TWR. The TWR increases by 3.5% by the side-wall secondary spark, as shown in Figure 9b.

Besides this use of a low conductive tool, the TWR is increased through the deposition of pyrolytic carbide onto the tool electrode. With an increase in the capacitance level, enhancing the hydrocarbon dielectric pyrolysis contributes to a simultaneous elevation in the TWR. The pyrolysis process deposits carbon on the µ-tool electrode, which decreases the tungsten carbide electrode’s heat conductivity and raises the TWR. The enormous plasma channels split into smaller, weaker ones due to the Al_2_O_3_ nano-powder. As a result, the speed of the chemical decomposition process (pyrolysis) decreases. It ultimately results in a decrease in the quantity of carbon deposited. The frequent impact of Al_2_O_3_ nanoparticles on the tool surface reduces the carbon deposition. When Al_2_O_3_ nano-powder is employed, the carbide layer’s thickness decreases, causing a reduction in the TWR.

### 3.4. Performance Analysis of Width of Machining

**Figure 10:**
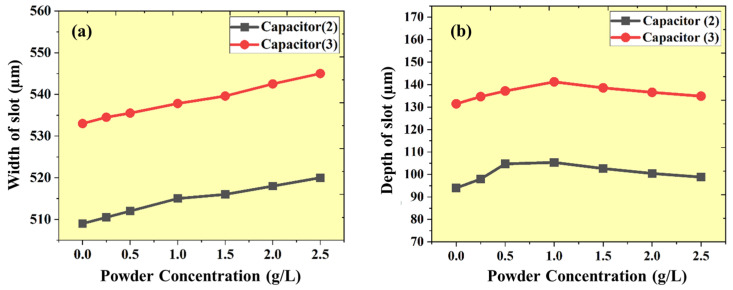
(**a**) Effect of Al_2_O_3_ nano-powder concentration on the width of generated micro-channel at different capacitors. (**b**) Effect of Al_2_O_3_ nano-powder concentration on the depth of generated micro-channel at different capacitors.

The experimental studies revealed that variables that affect the width of the micro-channel are the secondary spark at the side wall, the flush-out mass (debris and nano-powder), and the amount of MRR and TWR. The impact of the quantity of the Al_2_O_3_ nano-powder on the µ-channel width for various capacitor levels is depicted in Figure 10a. At the low capacitance level (100 pF) and using nano-powder, the particle concentration (debris and Al_2_O_3_) in the machining zone increases with a low gap voltage because multiple discharges at the side-wall interface of the tool and workpiece increase, which is the primary reason for enhancing the width. The width increased by 0.4% at each increment in the powder concentration. Similarly, at a high capacitance level (1000 pF), the effect of the nano-powder is enhanced in the form of uniform energy distribution with increasing the discharge points throughout the machining area, causing a side-wall sparking increase, followed by an enhanced width with a change of 0.3% at each increment in the powder concentration.

### 3.5. Performance Analysis of Depth of Machining

The micro-EDM technique with powder-mixed EDM oil generates the micro-channels and flushes out the removed material. Mixing the nano-powder increases the dielectric strength and flushing characteristics, directly impacting the micro-channels’ depth. At a high capacitance level (1000 pF), using nano-powder at 0.25 g/L concentration increases the depth by 2.4% because of enhancing the energy distribution, gap voltage, and flushing. Furthermore, for an increment in the concentration up to 1.0 g/L, the depth increased by 7.4%. After that, when the concentration further increased, the depth reduced by 2.0% because of unused nano-powder deposited on the surface and shorting and arcing. On the other hand, at a low capacitance level (100 pF), the same behavior as high capacitance is also shown. By using nano-powder at a 0.25 g/L concentration, the depth is increased by 4.2% because of enhanced gap voltage and flushing. Furthermore, with an increment in the concentration up to 1.0 g/L, the depth increased by 12%. After that, when the concentration was increased, the depth was reduced by 2.6% because of unused nano-powder deposited on the surface. Figure 10b demonstrates the impact of varying Al_2_O_3_ nano-powder concentrations on the micro-channel depth under different capacitors.

### 3.6. Implementation of DFA

DFA is used to concurrently optimize the responses to acquire the micro-channel’s nominal depth and width, maximum MRR, and minimum TWR. The researchers normalized their data and assessed individual desirability indexes from 0 to 1. The larger-is-better-type desirability function for MRR, the smaller-the-better-type for TWR and micro-channel width, and the nominal-is-better-type desire function for micro-channel depth. The desirability function (Equation (8)) defines the nominal-is-better-type quality feature, which is employed when a certain goal value is to be obtained. Here, y_min_ denotes the lesser value of y*, y_max_ represents the higher value of y*, y_target_ indicates the target value of y* and s, and t represents the weight allocated to responses.

(8) $$d_{i}=\left\{\begin{array}{c} {\left(\frac{y^{*}-y_{min}}{y_{target}-y_{min}}\right)}^{s},\;\;\;y_{min}\leq y^{*}\leq y_{target}\;,s\geq 0\\ {\;\left(\frac{y^{*}-y_{min}}{y_{target}-y_{min}}\right)}^{t},{\;\;\;y}_{min}\leq y^{*}\leq y_{max}\;,y_{target}\geq 0\\ 0 \end{array}\right.$$

The desirability function (Equation (9)) is utilized to define the larger-is-better quality characteristic when seeking to maximize the desired outcome. Here, y_min_ represents the lower value of y*, y_max_ represents the maximum value of y*, and r is the weight applied to replies.

(9) $$d_{i}=\left\{\begin{array}{c} 0\;,\;\;y^{*}\leq y_{min}\;\\ {\;\left(\frac{y^{*}-y_{min}}{y_{max}-y_{min}}\right)}^{r},{\;\;\;\;y}_{min}\leq y^{*}\leq y_{max}\;,r\geq 0\\ \;1\;,\;\;y^{*}\geq y_{max}\;\; \end{array}\right.$$

Equation (10) represents a “smaller-is-better” quality characteristic used for minimizing the answer. y_min_ represents the lower value of y*, y_max_ represents the highest value of y*, and r indicates the weight allocated to replies.

(10) $$d_{i}=\left\{\begin{array}{c} 1\;,\;\;y^{*}\leq y_{min}\;\\ {\;\left(\frac{y^{*}-y_{max}}{y_{min}-y_{max}}\right)}^{r},{\;\;\;\;y}_{min}\leq y^{*}\leq y_{max}\;,r\geq 0\\ \;0\;,\;\;y^{*}\geq y_{min}\;\; \end{array}\right.$$

After analyzing the individual desirability of the experimental values, the composite desirability is calculated as the geometric mean of the discrete desirability, as represented in Equation (11).

(11) $${CD}_{i}={\left(d_{1}*d_{2}*d_{3}*\ldots \ldots .*\;d_{n}\right)}^{\frac{1}{n}}={\left(\prod_{i=1}^{n}d_{i}\right)}^{\frac{1}{n}}$$

where *CD_i_* = composite desirability, and *n* = number of responses.

The individual desirability of the depth of the micro-channel, MRR, TWR, and width of the micro-channel is determined using desirability functions represented in Equations (12)–(15), respectively. Equal weightage (*n* = 4) is assigned to depth, MRR, width, and TWR.

(12) $$d_{depth}={\left(\frac{y^{*}-94}{110-y_{min}}\right)}^{0.25},\;\;\;94\leq y^{*}\leq 141.25\;,110\geq 0$$

(13) $$d_{mrr}=\left\{\begin{array}{c} \;\;\;\;\;\;\;\;\;\;\;\;0\;,\;\;{\;\;\;\;\;\;\;y}^{*}\leq 0.000632\\ {\;\left(\frac{y^{*}-0.000632}{0.003-0.000632}\right)}^{0.25},0.000632\leq y^{*}\leq 0.003\;\\ \;\;\;\;\;\;\;1\;,\;\;{\;\;\;\;\;\;y}^{*}\geq 0.003\;\; \end{array}\right.$$

(14) $$d_{twr}=\left\{\begin{array}{c} 1\;,\;\;y^{*}\leq 0.00016\;\\ {\;\left(\frac{y^{*}-0.000848}{0.00016-0.000848}\right)}^{0.25},0.00016\leq y^{*}\leq 0.000848\\ \;0\;,\;\;y^{*}\geq 0.000848 \end{array}\right.$$

(15) $$d_{width}=\left\{\begin{array}{c} 1\;,\;\;y^{*}\leq 509\;\\ {\;\left(\frac{y^{*}-545}{509-545}\right)}^{0.25},509\leq y^{*}\leq 545\;\\ \;0\;,\;\;y^{*}\geq 509\;\; \end{array}\right.$$

Equation (16) is used to calculate *CD_i_* based on the two replies under consideration.

(16) $${CD}_{i}={\left(d_{depth}*d_{mrr}*d_{twr}*d_{width}\right)}^{\frac{1}{4}}$$

Table 6 displays all experimental datasets’ derived desirability index and composite desirability (*CD_i_*) values. The maximum *CD_i_* achieved is 0.897499, and the discrete desirability indices for width, MRR, TWR, and depth are 0.955443, 0.754649, 0.980544, and 0.917739, respectively. The best optimum parameters, shown in bold, arise in experiment number 7 with a capacitor of 100 pF and a powder concentration of 1 g/L.

### 3.7. Effect of Powder Concentration and Energy Levels on Machined Surfaces

**Figure 11:**
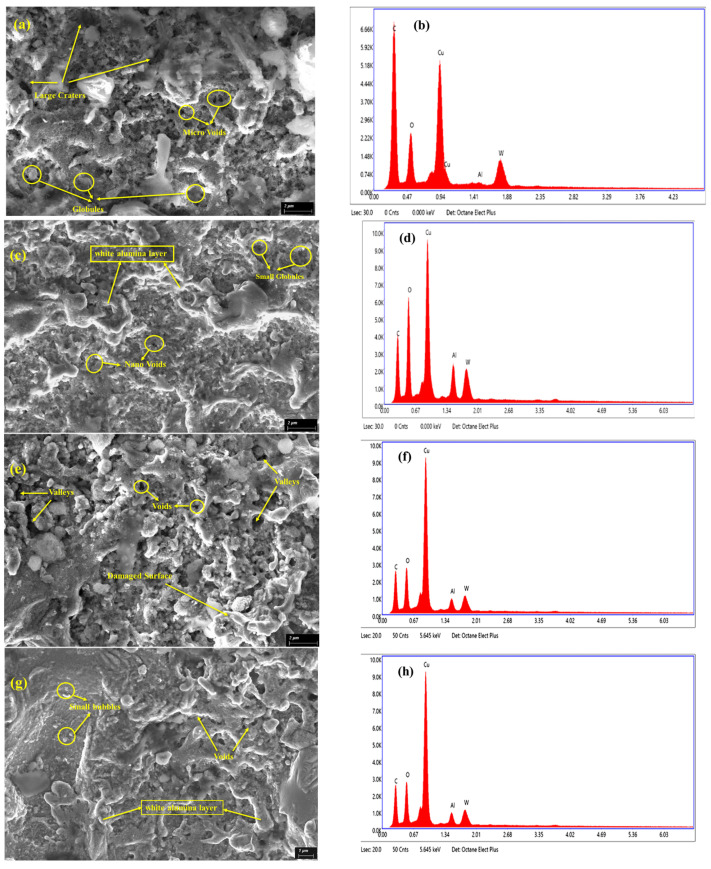
Surface morphology of µ-channels/slots at low energy level (100 pF) and different powder concentrations: (**a**) 0 g/L; (**b**) EDX image at 0 g/L; (**c**) 1 g/L; (**d**) EDX image at 1 g/L; and at high energy level (1000 pF) and different powder concentrations: (**e**) 0 g/L; (**f**) EDX image at 0 g/L; (**g**) 1 g/L; (**h**) EDX image at 1 g/L.

From previous discussion, it came to notice that each parameter impacts the MRR, TWR, and dimensions, including the machined surfaces. This section deals with the debate about machined surface analysis using EDX and SEM techniques. The result reveals that unevenly distributed surfaces are found at low capacitance levels (100 pF) without adding Al_2_O_3_ nano-powder, as shown in Figure 11a. This uneven distribution resulted in the creation of concentrated micro-voids and large craters in a random pattern due to the non-uniform energy distribution. However, a uniformly distributed surface was obtained with the addition of alumina nano-powder (1.0 g/L) in dielectric due to a reduction in the size of the voids and globules up to the nano-scale, as shown in Figure 11c. Moreover, the inclusion of alumina nano-powders ensures a uniform distribution of powder particles between the cathode and anode, resulting in enhanced surface quality. However, when the capacitance is low (100 pF), the debris, consisting of both the parent material and nano-powder, tends to accumulate on the machined surface due to the insufficient energy and time for evacuation from the machining zone. This phenomenon is also reflected in the EDX analysis, which shows a significant increase in the percentage of aluminum, as depicted in Figure 11b,d. As the energy level increases to 1000 PF, more surface damage is caused without the use of alumina nano-powder. The damage manifests as valleys, large voids, and an unevenly distributed damaged surface throughout the machining surface, as illustrated in Figure 11e. Figure 11f displays EDX studies of a micro-channel at a high energy level and a concentration of 0 g/L powder. Adding alumina nano-powder in dielectric fluid improves the machined surface quality, resulting in fewer voids and smoother surfaces, as illustrated in Figure 11g. However, with increasing the energy, the gap voltage also increases, making eliminating debris from the machining area easier, and the longer cooling time prevents debris accumulation. The EDX analysis in Figure 11h indicates a 49.70% decrease in the percentage accumulation of alumina nano-powder.

Consequently, to achieve the desired output of an efficient process and surface modification, the recast layer thickness could be lowered when operating at high capacitance. Therefore, selecting the appropriate capacitance level, whether low or high, is essential based on the desired output.

## 4. Conclusions

The current research explores how the presence of Al_2_O_3_ nano-powder and its varying concentration affect the process of micro-electro discharge milling on copper, considering two different energy regimes. The dimensions (width and depth) of the micro-channels on the machined samples, as well as TWR and MRR, are among the characteristics used in the study to evaluate the milling process’s performance. The goal of the DFA was to determine the ideal set of parameters for each outcome that was measured. The following observations are derived from the experimental findings:

- The nano-powder shows different behaviors at different capacitance levels. At low capacitance, it enhances gap voltage and flushing. On the other side, at high capacitance, it enhances energy distribution.
- The nano-powder also improves the machining performances in terms of MRR, which increased by 50.5% (100 pF) and 8.7% (1000 pF); TWR was reduced by 4.2% (100 pF) and 3.3% (1000 pF), the width increased by 0.4% (100 pF) and 0.3% (1000 pF), and the depth increased by 12% (100 pF) and 7.4% (1000 pF).
- The nano-powder optimum concentration depends on the energy levels at which the desired objective is achieved. At low capacitance, it is 0.8 g/L, and at high capacitance, it is about 1.0 g/L. Excess concentrations act like non-energized debris, which degraded the machining performance.
- At low capacitance (100 pF), the machined surface was coated with a mass (debris + nano-powder), which enhanced the surface quality of the material. But, at high capacitance (1000 pF), the surface deposition is low, and the dimensional accuracy is high.
- As per the desirability (maximum MRR, targeted depth, minimum TWR, and width), 1.0 g/L of nano-powder concentration showed the best performance at a low capacitance level (100 pF).

Powder-mixed micro-EDM shows significant potential for enhancing the surface finish, dimensional accuracy, and machinability of hard-to-machine materials, with applications in microfluidics, biomedical devices, aerospace, and electronics. It also addresses the growing need for miniaturized components, machining of advanced materials, and achieving high aspect ratios with improved productivity. Future studies should focus on exploring various nano-powders, optimizing process parameters, and using advanced surface characterization techniques. Hybridizing micro-EDM with methods like laser machining, micro-ECM, vibration-assisted, or magnetic field-assisted machining can further improve the overall efficiency and surface quality. However, scaling the process for industrial use presents challenges, particularly due to nano-powder agglomeration in the dielectric fluid, which can affect the discharge stability and machining consistency. Techniques such as ultrasonic-assisted mixing or sonication can enhance dispersion, but prolonged mixing or storage may alter the powder’s properties, reducing the process reliability. Additionally, the effects of side sparking on localized heating and surface integrity require further investigation to ensure consistent performance in large-scale applications.

## Figures and Tables

**Table 1:** Properties of tool electrode and workpiece materials.

Properties	Workpiece (Cu)	Electrode (WC)
Composition (wt.%)	Pure Cu	90% W
Density (g/cm^3^; at 25°C)	8.96	15.63
Melting Point (°C)	1083	2870
Thermal Conductivity [W/mK]	401	110
Thermal Expansion Coefficient (K^−1^; 0–100 °C)	16–16.7 × 10^−6^	4.5–6.0 × 10^−6^
Electrical Resistivity (Ωm)	1.72 × 10^−8^	65 × 10^−6^
Size	Thickness: 2 mm	Diameter: 500 µm
Hardness (HV)	100	1300
Tensile Strength (MPa)	210	344

**Table 2:** Parameters and their levels in machine processes.

Features	Specifications
Machine Movement (mm)	Maximum Movement: 135 × 65 × 65
Travel Accuracy (µm)	Precision: 5, Recurrence: 2
Energy Level (pF)	0, 33, 100, 1000, 10,000
Voltage (V)	24 to 200
Feed (µm/s)	5 to 15
Polarity	Forward and Reverse

**Table 3:** Experimental details, including process parameters and their levels.

Working Condition	S.I Unit	Description
Voltage	V	115
Energy Level (Capacitance)	pF	2 (100), 3 (1000)
Powder Specification	Nm	Al_2_O_3_, Shape—Spherical, Size—25
Powder Concentration	g/L	0, 0.25, 0.5, 1.0, 1.5, 2.0, 2.5
Tool Travel Speed	µm/s	8
Length of Slot/Channel	mm	2.5
Depth of Slot/Channel	µm	110
Dielectric Fluid	**-**	Micro-EDM oil
Polarity	**-**	Reverse; Workpiece [Cathode(−ve)], Tool [Anode(+ve)]

**Table 4:** Experimental outcome data of width, depth, MRR, and TWR.

Exp. No.	Capacitor	Powder Concentration (g/L)	Width of Micro-Channel (µm)	Depth of Micro-Channel (µm)	TWR (mm^3^/min)	MRR (mm^3^/min)
1.	2	0	509	94	0.00017	0.00063
2.	3	0	533	131.45	0.00085	0.00230
3.	2	0.25	510.5	98	0.00016	0.00095
4.	3	0.25	534.5	134.64	0.00083	0.0025
5.	2	0.5	512	104.74	0.00020	0.0012
6.	3	0.5	535.5	137.135	0.00081	0.0027
7.	2	1.0	515	105.35	0.00021	0.0014
8.	3	1.0	537.8	141.25	0.00079	0.0030
9.	2	1.5	516	102.65	0.00023	0.0012
10.	3	1.5	539.6	138.546	0.00082	0.0028
11.	2	2.0	518	100.45	0.00023	0.0001
12.	3	2.0	542.5	136.564	0.00083	0.0027
13.	2	2.5	520	98.85	0.00020	0.0001
14.	3	2.5	545	134.854	0.00084	0.0025

**Table 5:** Two-stage nested analysis of MRR, TWR, width, and depth.

Control Parameter’s	DOF	Seq. SS	Adj. MS	F-Value	%PC	Remarks
NESTED ANOVA for MRR						
Powder Concentration (A)	1	1 × 10^−6^	1.3 × 10^−6^	0.87	6.77	
Capacitor (B) (Within Powder Concentration)	12	1.8 × 10^−5^	1.5 × 10^−6^	637.74	93.06	Significant
Error	14	0.00	0.00			
Total	27	2.0 × 10^−5^				
NESTED ANOVA for TWR						
Powder Concentration (A)	1	4.4 × 10^−9^	4.4 × 10^−9^	1.92 × 10^−2^	0.16	
Capacitor (B) (Within Powder Concentration)	12	2.73 × 10^−6^	2.28 × 10^−7^	1.05 × 10^4^	99.83	Significant
Error	14	3.0 × 10^−10^	2.0 × 10^−11^			
Total	27	2.74 × 10^−6^				
NESTED ANOVA for Width						
Powder Concentration (A)	1	416.42	416.42	1.24	9.36	
Capacitor (B) (Within Powder Concentration)	12	4030.05	335.84	1741.38	90.6	Significant
Error	14	2.70	0.19			
Total	27	4449.17				
NESTED ANOVA for Depth						
Powder Concentration (A)	1	337.68	337.68	0.45	3.63	
Capacitor (B) (Within Powder Concentration)	12	8967.95	747.33	1.51 × 10^4^	96.4	Significant
Error	14	0.69	0.05			
Total	27	9306.32				

**Table 6:** Estimated desirability values.

Expt. No	Capacitor	Powder Conc. (g/L)	Desirability Index				Composite Desirability (CD_i_)	Rank
			Width	TWR	MRR	Depth		
1	2	0	1	0.99745	0	0	0	12
2	3	0	0.75984	0	0.91612	1.23690	0	12
3	2	0.25	0.98942	1	0.60536	0.70711	0.80671	6
4	3	0.25	0.73490	0.41292	0.94243	1.26243	0.77515	9
5	2	0.5	0.97848	0.98552	0.69983	0.90515	0.88406	2
6	3	0.5	0.71673	0.47492	0.96080	1.28138	0.80459	7
7	2	1	0.95544	0.98054	0.75465	0.91774	0.89750	1
8	3	1	0.66874	0.53884	1	1.31090	0.82903	4
9	2	1.5	0.94738	0.97432	0.68389	0.8575	0.85775	3
10	3	1.5	0.62233	0.45697	0.97818	1.29173	0.77424	10
11	2	2	0.93061	0.97234	0.61916	0.796819	0.817404	5
12	3	2	0.51335	0.40218	0.96670	1.277117	0.71054	11
13	2	2.5	0.91287	0.98589	0.56888	0.74200	0.78508	8
14	3	2.5	0	0.37076	0.94243	1.26409	0	12

## Data Availability

The authors have mentioned that all of the necessary data and code to replicate the research can be found within the main text of the paper.

## Abbreviations

The following abbreviations are used in this manuscript:

P_c_	Powder concentration [g/L]
V	Voltage [volts]
ρ	Density [gm/mm^3^]
w_i_ and w_f_	Initial and final weights [gram]
MRR	Material removal rate [mm^3^/s]
PC	Percentage contribution [%]
TWR	Tool wear rate [mm^3^/s]
mm	Millimeter
nm	Nanometer
pF	Pico faraday
μm	Micrometer
μm/s	Micrometer per second
μ-EDM	Micro-electrical discharge machining
ANOVA	Analysis of variance
Adj. MS	Adjusted mean squares
CD_i_	Composite desirability
DOF	Degree of freedom
DI	Deionized
EDM	Electrical discharge machining
DFA	Desirability function analysis
EDX	Energy dispersive X-ray
g/L	Gram per liter
MEMS	Micro-electromechanical systems
NPMEDM	Nano-powder mixed electric discharge machine
PM μ-EDM	Powder-mixed micro-electrical discharge machining
Seq. SS	Sequential sum of squares
SEM	Scanning electron microscope
